# A new colorimetric DPPH^•^ scavenging activity method with no need for a spectrophotometer applied on synthetic and natural antioxidants and medicinal herbs

**DOI:** 10.1080/14756366.2017.1284068

**Published:** 2017-03-06

**Authors:** Zeynep Akar, Murat Küçük, Hacer Doğan

**Affiliations:** a Department of Genetics and Bioengineering, Faculty of Engineering and Natural Sciences, Gumushane University, Gumushane, Turkey;; b Department of Chemistry, Faculty of Sciences, Karadeniz Technical University, Trabzon, Turkey;; c Faculty of Engineering and Natural Sciences, Gumushane University, Gumushane, Turkey

**Keywords:** TLC plates, Image J software, 2,2-diphenyl-1-picrylhydrazyl, antioxidant

## Abstract

2,2-Diphenyl-1-picrylhydrazyl (DPPH^•^) radical scavenging, the most commonly used antioxidant method with more than seventeen thousand articles cited, is very practical; however, as with most assays, it has the major disadvantage of dependence on a spectrophotometer. To overcome this drawback, the colorimetric determination of the antioxidant activity using a scanner and freely available Image J software was developed. In this new method, the mixtures of solutions of DPPH^•^ and standard antioxidants or extracts of common medicinal herbs were dropped onto TLC plates, after an incubation period. The spot images were evaluated with Image J software to determine CSC_50_ values, the sample concentrations providing 50% colour reduction, which were very similar with the SC_50_ values obtained with spectrophotometric method. The advantages of the new method are the use of lower amounts of reagents and solvents, no need for costly spectrophotometers, and thus significantly lowered costs, and convenient implementation in any environment and situation.

## Introduction

Oxidation is a chemical reaction that transfers hydrogen or electrons from a substance to an oxidising agent, and oxidation reactions can generate free radicals. Free radicals are also produced as a consequence of the incomplete reduction of oxygen molecule^1^. The free radicals formed can cause structural and functional changes in biomolecules. Antioxidants protect biological systems against free radical damage. Insufficient levels of endogenous and exogenous antioxidants can cause oxidative stress, an imbalance of oxidants and antioxidants resulting in cellular damage or death. Oxidative stress plays a significant role in diverse diseases such as cardiovascular conditions, cancer, inflammatory diseases and early ageing (progeria)^2^^,^^3^. Antioxidant components of plants are also effective in preventing many diseases^4^.

Secondary metabolites are organic compounds that are not directly involved in growth and development of an organism and play an important role in plant defence. They are used as a raw material in many industries e.g. food antioxidants, antimicrobials, and pharmaceuticals. One of the largest class of plant secondary metabolites is phenolics. Phenolics serve in defence by scavenging or preventing the formation of reactive oxygen species (ROS) to avoid molecular damage and other damaging factors. Plants contain various types of compounds with antioxidant activities, such as antioxidant vitamins (A, C, and E), carotenoids, coenzyme-Q, lycopenes, and phenolics (phenolic acids, flavonoids, flavonols, anthocyanins, tannins, and lignins)^5^.

The antioxidant activity of natural and synthetic compounds and extracts of various natural sources including medicinal herbs are determined using various antioxidant methodologies^6–9^. Antioxidant assays may be classified based on the type of antioxidants measured (lipophilic or hydrophilic, enzymatic or non-enzymatic), character of solvent (aqueous or organic), type of reagent (radicalic or non-radicalic), and mechanism of reaction (hydrogen atom transfer, HAT; electron transfer, ET)^10^. ET-based assays include 2,2-diphenyl-1-picrylhydrazyl (DPPH^•^) scavenging^11^, ferric reducing/antioxidant power (FRAP)^12^, 2,2-azinobis-(3-ethylbenzothiazoline-6-sulfonic acid) (ABTS•+) scavenging^13^, cupric ion reducing antioxidant capacity (CUPRAC), and total fenolic content with folin ciocalteu reagent (FCR). The examples for the methods based on HAT are oxygen radical absorbance capacity (ORAC) and total radical absorption potentials (TRAP).

DPPH^•^ assay is one of the most popular and frequently employed method among antioxidant assays. The method is simple, efficient, relatively inexpensive, and quick. However, as with most antioxidant assays, it requires a UV–Vis spectrophotometer. DPPH^•^ method was developed by Blois^14^ and modified by Brand-Williams et al.^11^ to produce the current widely used form. DPPH^•^ is a stable free radical which possesses a deep purple colour and a strong absorption around 517 nm. The antioxidant compounds present in the medium convert DPPH^•^ radical to a more stable DPPH^•^ molecular product by donating an electron or a hydrogen atom. The colour change from purple of DPPH^•^ radical to pale yellow of reduced form of DPPH^•^ allows the spectrophotometric determination of the antioxidant activity. The results are either expressed as SC_50_ (otherwise called the IC_50_ value), the concentration of the antioxidant causing 50% DPPH^•^ scavenging^15^^,^^16^, or as %scavenging of DPPH^•^ at a fixed antioxidant concentration for all the samples.

DPPH^•^ spectrophotometric method has lately been utilised in on-line HPLC systems to determine individual antioxidant components^17^^,^^18^. Although spectrophotometric DPPH^•^ method has found many applications in various fields, it has the major disadvantage of requiring spectrophotometer and thus high costs.

In the search for the reduced costs for bioactivity testings, new strategies have been developed. For such purposes, biological activity studies are made using flat surfaces, such as TLC plates or filter papers, to make substitution possible for spectrophotometers^19^. Studies that determine the antioxidant activity using TLC plates are available with names bioautography, spot assay etc^20^. In bioautographic applications, plant extracts have been applied as spots on TLC plates or chromatography papers, and the plates/papers have been immersed in the solvent tank containing mobile phase solvent mixtures^21^^,^^22^. The antioxidant activities of the components thus separated were screened for their antioxidant activity based on DPPH^•^ radical scavenging methodology by spraying DPPH^•^ solution onto the TLC plate or immersing the plate into DPPH^•^ solution and observing the colour pattern^23^^,^^24^.

In recent years, the images formed in various studies have been transferred to a computer and evaluated using suitable image processing software^25^. In one example, phenolic compounds dissolved in water were analysed on a molecularly imprinted polymer (MIP) membrane^26^. In another colorimetric investigation, phenolic compounds such as phenol, bisphenol A, catechol, and cresols were applied onto the paper which contain tyrosinase enzyme sprayed previously. The concentration of the phenolics and colour values were found to increase proportionally^27^.

Many programmes have been developed to analyse the images transmitted to the computer. Besides very expensive programmes, readily available free image processing software can be found on the Internet. Image J, the free software used in the current study, is utilised in many disciplines and allows the analysis of a diversity of samples in different areas^28–30^. In addition, enzymatic phenol analysis was made by dropping on filter paper using this programme^27^^,^^31^.

In this study, a new colorimetric DPPH^•^ test with the materials easily found in any laboratory without the need for a spectrophotometer was developed. The new method aims at the use of lower amounts of reagents and solvents, the elimination of the need for costly spectrophotometric devices, and thus lower costs, and less dependence on electric devices. No DPPH^•^ test made by dropwise application of reaction mixture over a flat medium and determination of antioxidant activity based on the colour value is found in the literature. DPPH^•^ antioxidant activity test is more practical with the newly developed method.

## Materials and methods

### Plant materials and chemicals/reagents

The dry plants green tea, alkanet, tilia, turmeric, rosemary, mint, hypericum perforatum and *Aloe vera* were purchased from a local herbal store in Trabzon, Turkey. HPLC-grade ethanol, methanol, and ethyl acetate were purchased from Merck. DPPH^•^ radical was obtained from Sigma. TLC silica gel 60 plates were purchased from Merck. Catechine was purchased from Aldrich, and ferulic acid was purchased from Fluka. Gallic acid, caffeic acid, syringic acid, quercetin, protocatechuic acid, BHT, chlorogenic acid, rutin, and all the other standard phenolics were purchased from Sigma.

### Plant extraction

Plants were dried at 50 °C for 48 h. Dry plant samples were extracted as follows: 10 g of plant material was extracted with 100 mL extraction solvent on a magnetic stirrer for 2 h. At the end of the extraction process, the extracts were filtered sequentially with filter paper and syringe filter (Minisart, NY 0.45 μm). The clear solutions were kept at room temperature and in a cool and dark environment till test time.

### Plant component determination

Plant extracts were injected to an Agilent 1200 HPLC instrument equipped with a diode array detector (DAD) for the determination of phenolic composition using 22 phenolic standards together with ascorbic acid. The separation of phenolics was carried on a Luna C18 column (25 cm × 3.00 mm i.d., 5 μm particles). The mobile phase consisted of 3% acetic acid in water (solvent A) and 0.5% acetic acid in acetonitrile:water (1:1) (solvent B) The following optimised gradient programme was used: 0–5 min (5% B), 5–20 min (15% B), 20–30 min (15% B), 30–55 min (80% B), 55–56 min (100% B), 56–60 min (100% B), before returning to the initial conditions. The flow rate was 0.6 mL/min, and the detection was made at 280 nm. Compound identification was based on retention time and UV spectra.

### Conventional spectrophotometric DPPH^•^ radical scavenging activity method

The radical scavenging activity was tested by utilising the widely used DPPH^•^ radical. 750 μL plant extracts and standard antioxidant samples of various concentrations with a two-fold serial dilutions were added to 750 μL of 2000 μM methanolic DPPH^•^ solution. The contents were then vortex-mixed and incubated for 60 min at room temperature. After the mixtures diluted at a 1/20 ratio, the absorbance values were determined using a UV–Vis spectrophotometer (ATI/Unicam UV2). The absorbances measured at 517 nm were plotted against sample concentration, and SC_50_ values were determined from the graphs as the sample concentration reducing DPPH^•^ concentration to half of its initial value. Reagent blank and solvent control tests were also made, and the results were used in the construction of the graphs. Lower SC_50_ values indicate higher radical scavenging potential.

### New dropping DPPH^•^ radical scavenging activity method

For the new dropping DPPH^•^ test, a pre-test was conducted for standards and plant extracts to determine approximate working concentration range. The mixing of test samples and DPPH^•^ solution and incubation were done as in spectrophotometric method. At the end of incubation, 15 μL of the mixtures were dropped onto a TLC plate (Merck Silica gel 60) as triplicates. After 5 min incubation, TLC plates were scanned (HP Deskjet Ink Advantage 2060 printer) by adjusting colour settings (brightness −100, contrast 85). The images were saved as jpeg files. The colour value of the each spot on TLC plates was determined as mean grey value by “Image J” software. The colour values were plotted against sample concentrations for the calculation of CSC_50_ values. CSC_50_ is the concentration of the sample which increases the colour value (intensity of colour) to half of its maximum at complete DPPH^•^ reduction. The 1st degree derivative graphics were also prepared by plotting (Δ_colour value/_Δ_concentration_) against concentration. CSC_50_ obtained from the derivative graph is the sample concentration showing the highest Δ_colour value/_Δ_concentration_ value.

The new method was optimised to achieve repeatable results. The optimised forms of the test working concentration by evaluating 50–2000 μM concentrations of DPPH^•^ radical, the drop volume by testing 10–100 μL range and the reaction environment (on TLC plate and in test tube) were determined. Furthermore, kinetic studies were performed to determine the reaction time with the standard antioxidants. The optimised conditions were used in the tests with plant extracts and standard antioxidants.

### Validation and statistical analysis

The curves for SC_50_ or CSC_50_ value calculations were constructed by running standards or plant extracts of five different concentrations, in triplicate. The similarity for 50% DPPH^•^ scavenging concentrations of the standards and samples between conventional method and the new method was determined with an R^2^ value of 0.9923. Limit of detection (LOD), limit of quantitation (LOQ), and coefficient of variation (CV) values were determined for the newly developed method comparatively with the conventional spectrophotometric method.

## Results and discussion

There are a number of antioxidant activity assays in the literature with different methodologies. DPPH^•^ radical scavenging activity is the most commonly used method to determine the antioxidant activity of natural and synthetic materials^9^^,^^32^ because it is quick and simple. Through the developments the method was transformed to be suitable in on-line HPLC-DPPH studies by using additional pumps and detectors in the HPLC systems^18^. As expected the coverage and costs of the used devices increased with method development. Thus, in recent years various strategies have been started to be established to reduce the costs in bioactivity studies as in DPPH^•^ antiradical investigations^23^.

As with the most antioxidant assays, DPPH^•^ method requires a spectrophotometer to measure absorbance at 517 nm. When antioxidant samples are mixed with DPPH^•^ reagent solution, the colour is turned to yellow from purple by time. The colour change is determined by measuring absorbance with a spectrophotometer at 517 nm. Even though this method is considered very simple and efficient, it does present various limitations. One of the most important limitations of conventional DPPH^•^ method is the requirement of a spectrophotometer. In the new method, colour change is evaluated by scanning the image and using free image processing software (Image J) without the use of a spectrophotometer.

In this study, a new DPPH^•^ assay was developed as more convenient than traditional methods with the major advantage of no spectrophotometer requirement. Colour measurement was made by using a smooth surface (TLC or paper), a scanner and the free downloadable colour measurement software Image J. The other advantages of the new method are consumption of less chemicals (reagent and solvent) and reduction of total test time. Another advantage is the much lower costs of the new method as no plastic cuvettes and washing solvent consumption are needed as opposed to the classical method.

**Figure 1.**
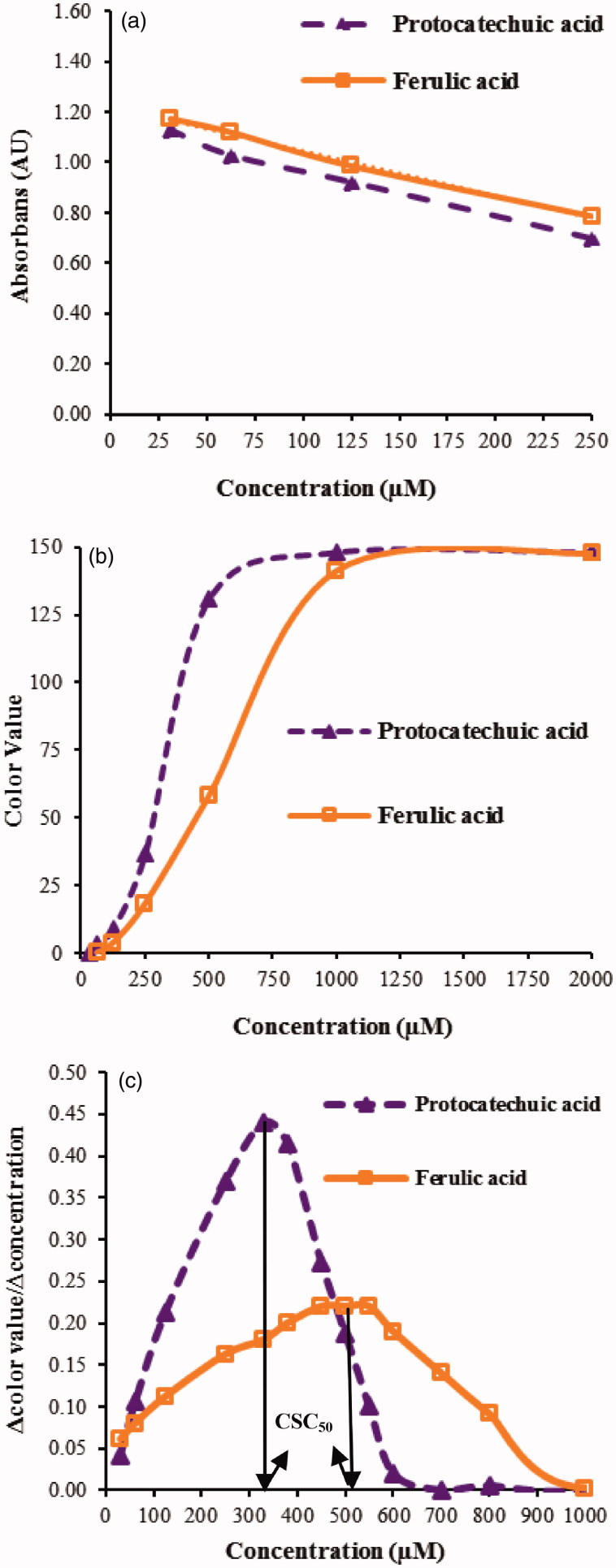
(a) Concentration–absorbance graph used to calculate SC_50,_ (b) concentration–colour value graph used to calculate CSC_50_ and (c) 1st degree derivative graph used to calculate CSC_50_ for protocatechuic acid and ferulic acid standards.

In the current study, SC_50_ value for each standard and sample was determined by applying the conventional method (Figure 1(a)). SC_50_ values were the sample concentration reducing DPPH^•^ concentration to half of its initial value, and thus the initial absorbance. The antioxidant capacities of standards and samples were determined as CSC_50_ values in the new DPPH^•^ method (Figure 1(b)). CSC_50_ is the concentration of the sample which increases the colour value (mean grey value) to half of its maximum observed at complete DPPH^•^ reduction. The derivative graphs were also prepared by using the colour values and sample concentrations (Figure 1(c)). The CSC_50_ values obtained with the derivative method were observed to be more compatible with the SC_50_ values in comparison to the CSC_50_ values obtained directly from colour values vs. sample concentration graphs. Therefore, the results of the further experiments were evaluated as CSC_50_ values for each standard and sample based on the graph prepared by plotting 1st degree derivative of colour value with respect to sample concentration (Δ_colour value/_Δ_concentration_) versus sample concentration (Figure 1(c)).

**Figure 2.**
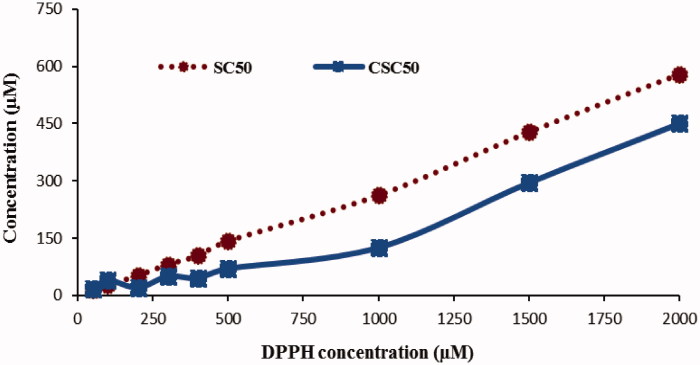
Linearity of SC_50_ and CSC_50_ values as a function of DPPH• radical concentration using Trolox standard.

In the development of the method, the DPPH^•^ concentration was first optimised by using Trolox standard at different concentrations to determine SC_50_ and CSC_50_ values with classical and the new methods, respectively. DPPH^•^ concentration was studied between 50–2000 μM. The concentration range of 1000 to 2000 was found to show good correlation between the two methods, and 2000 μM was chosen for later tests for the production of more dependable colour measurements (Figure 2).

**Figure 3.**
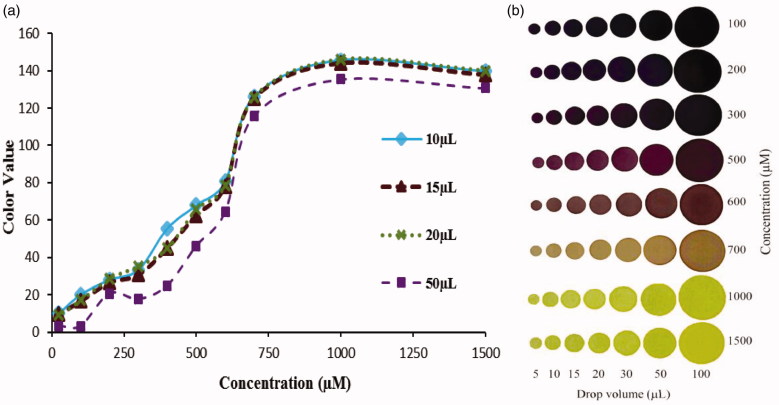
The effect of drop volume on colour value readings using Trolox standard.

To determine the appropriate dropping volume, experiments were made with Trolox standard of different volumes (10–100 μL) and concentrations (100–1500 μM) by using a 100 μL micropipette with a fixed 2000 μM concentration of DPPH^•^. Although 10, 15 and 20 μL were observed to be suitable drop volumes (Figure 3), volumes of larger than 10 μL are required to provide free dropping of the mixture from the pipette tip. Otherwise pipette tip has to be touched to the surface, which may affect the colour readings. It has been decided that the appropriate value for dropping volume are 15 or 20 μL. In order to lower the consumption of solvents and samples, the studies were done with 15 μL sample volume. Above 20 μL volume, the colour readings and thus the graphs appeared to be non-linear (Figure 3).

**Figure 4.**
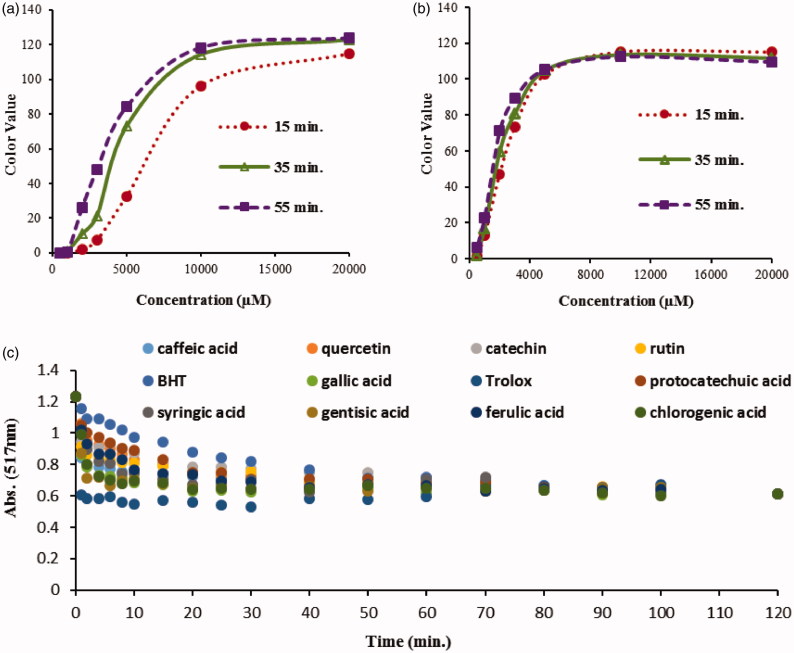
The effect of incubating the reaction mixtures in (a) test tubes and (b) on TLC for 55 min using shringaldehyde as standard, and (c) trends in absorbance decrease as DPPH was scavenged with the standards as a function of time, i.e. reaction kinetics.

The reaction between antioxidant syringaldehyde and DPPH^•^ radical was followed by performing the incubation phase in test tube (Figure 4(a)) or as drops on TLC plate (Figure 4(b)). When the drop colour value was followed on the plate for 55 min to monitor reaction progression, not much difference was observed between the CSC_50_ values, which shows inefficiency of this application in presenting the reaction kinetics. On the other hand, the colour values determined for the drops prepared after the incubation in the tubes provided relatively different CSC_50_ values as would be expected because of time dependence of DPPH^•^ – antioxidant reaction. Thus, it is decided that the colour readings on TLC plates should be done after incubation has been completed in test tubes.

To study the completeness of the reaction, 12 standards were tested by following the reactions during an incubation period of 120 min measuring absorbance at 517 nm with a spectrophotometer (Figure 4(c)). The incubation time for further tests was decided to be 60 min. Results were found to be consistent with the incubation time (30–60 min) in the literature^33^^,^^34^.

The new method was tested for validation parameters of limit of detection (LOD), limit of quantitation (LOQ) and coefficient of variation (CV) in comparison to the conventional spectrophotometric method (Table 1). As expected, the classical method with the use of more sophisticated instrument showed better results. The new colorimetric method showed well acceptable LOD, LOQ and CV values especially for screening applications.

**Table 1.** Validation parameters of spectrophotometric and the new colorimetric methods.

	LOD (μM)		LOQ (μM)		CV^a^	
	Spect.	Color.	Spect.	Color.	Spect.	Color.
Gallic acid	0.67	4.50	2.22	11.67	0.078	1.198
Caffeic acid	1.11	4.32	3.69	14.41	0.090	0.929
BHT	2.09	6.80	6.96	22.65	0.111	1.214

a Coefficient of variation (CV) values are based on absorbance and colour values.

The plants used in this study were selected from plants based on literature report^32^ and our previous preliminary investigation. The selected plants have different antioxidant capacities from high through medium to low degrees. Various solvent extracts of these plants were tested. The phenolic composition of the methanolic plant extracts were determined using 22 phenolic acids and flavonoids (Table 2). The extracts included few of the standards. Gallic acid and protocatechualdehyde were present in the three samples. Mint and tilia samples appeared to contain highest numbers of the standards used.

**Table 2.** Phenolic components of the plant methanolic extracts.

	Concentration (mg/L)								
	Mint	Turmeric	*H. perforatum*	G. tea	Rosemary	Alkanet	Tilia	*A. vera*	Equation Area = a × Amount ± b
Ascorbic acid	n.d.	n.d.	n.d.	n.d.	n.d.	n.d.	n.d.	n.d.	y = 59798.x + 4024.3
3,4-Dihydroxybenzoic acid	n.d.	n.d.	n.d.	n.d.	n.d.	n.d.	n.d.	n.d.	y = 176,94.x − 2,53
Benzoic acid	4.27	n.d.	n.d.	n.d.	n.d.	n.d.	n.d.	n.d.	y = 30,02.x + 6,47
Caffeic acid	n.d.	n.d.	n.d.	n.d.	n.d.	n.d.	n.d.	n.d.	y = 359,46.x + 2,525
Catechin	n.d.	n.d.	n.d.	n.d.	n.d.	n.d.	n.d.	n.d.	y = 81,32.x − 13,601
Chlorogenic acid	30.82	n.d.	n.d.	n.d.	25.60	n.d.	n.d.	n.d.	y = 135,34.x + 13,63
Ferulic acid	n.d.	n.d.	17.65	n.d.	n.d.	n.d.	n.d.	n.d.	y = 301,46.x + 61,47
Flavone	n.d.	n.d.	n.d.	n.d.	n.d.	n.d.	n.d.	n.d.	y = 0,038.x + 0,864
Galangin	n.d.	n.d.	n.d.	n.d.	n.d.	n.d.	n.d.	n.d.	y = 0,058.x + 0,098
Gallicacid	0.19	n.d.	n.d.	37.32	n.d.	n.d.	0.99	n.d.	y = 444,92 x + 35,251
Myricetin	n.d.	n.d.	n.d.	n.d.	n.d.	n.d.	n.d.	n.d.	y = 56,26.x − 59,51
p-Coumaric acid	n.d.	n.d.	5.09	n.d.	n.d.	n.d.	n.d.	n.d.	y = 607,82.x − 8,11
p-OH Benzoic acid	n.d.	n.d.	n.d.	n.d.	n.d.	n.d.	n.d.	n.d.	y = 179,22.x + 0,84
Protocatechualdehyde	0.72	n.d.	n.d.	n.d.	1.03	n.d.	0.51	n.d.	y = 526,32.x + 4,96
Protocatechuic acid	n.d.	3.05	n.d.	n.d.	n.d.	n.d.	8.01	n.d.	y = 161,57.x − 5,45
Quercetin	n.d.	n.d.	n.d.	n.d.	n.d.	n.d.	n.d.	n.d.	y = 27,02.x + 0,9
Rutin hydrate	1.78	n.d.	n.d.	n.d.	n.d.	n.d.	77.80	n.d.	y = 73,88.x − 0,57
Sesamol	n.d.	n.d.	n.d.	n.d.	n.d.	n.d.	n.d.	n.d.	y = 88,92x + 0,948
Sinapic acid	n.d.	n.d.	n.d.	n.d.	n.d.	n.d.	0.70	n.d.	y = 176,96.x + 3,54
Syringaldehyde	n.d.	n.d.	n.d.	31.50	n.d.	n.d.	n.d.	n.d.	y = 254,95.x − 478
Syringic acid	n.d.	n.d.	n.d.	n.d.	n.d.	n.d.	0.70	n.d.	y = 453,2.x + 3,147
Vanillic acid	n.d.	n.d.	n.d.	n.d.	n.d.	n.d.	n.d.	n.d.	y = 221.x + 3,26
Vanillin	n.d.	n.d.	7.06	n.d.	n.d.	n.d.	n.d.	n.d.	y = 499,2.x + 1,86

n.d.: Not detected (below quantification limit or unobserved).

**Figure 5.**
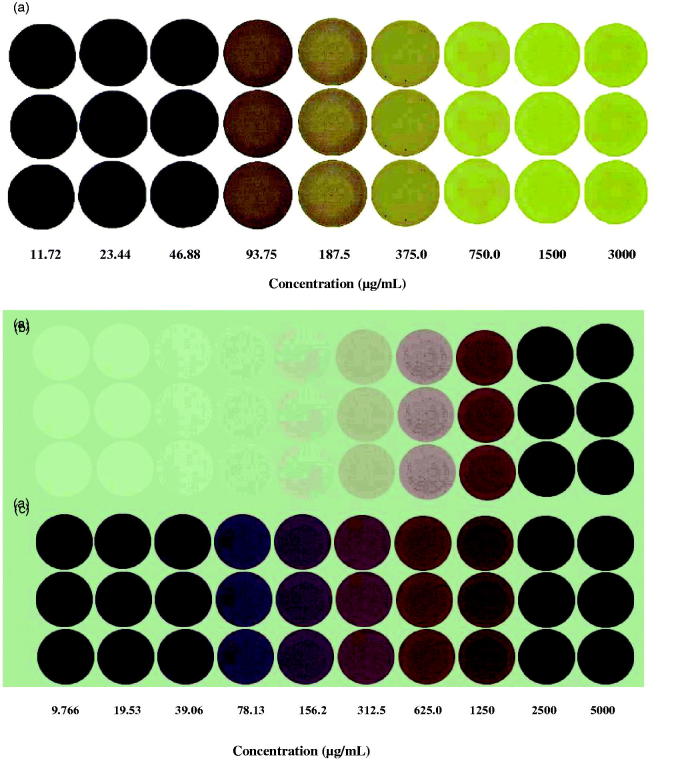
(a) Image of spots prepared with 1:1 serial dilution of gallic acid beginning from 3000 μg/mL highest concentration, and image of spots prepared with 1:1 serial dilution of alkanet extract beginning from 5000 μg/mL highest concentration containing (b) extract and reagent solvent (methanol) and (c) extract and DPPH^•^ reagent.

The DPPH^•^ reaction mixtures of all of the standards and the samples were dropped on TLC plates as three parallel and at different concentrations. The colour values were determined with image J software. Gallic acid was serially diluted 1:1 starting from 3000 μM for nine concentrations (Figure 5(a)). Alkanet sample was applied with 1:1 dilution starting from 5 mg/mL (Figure 5(b,c)). In this study, because standard antioxidants and the reagent solvent are colourless, blank test could not be done. But the colour value for the blank test for plant extracts was determined using the drops that contain only the sample and DPPH^•^ solvent.

**Figure 6.**
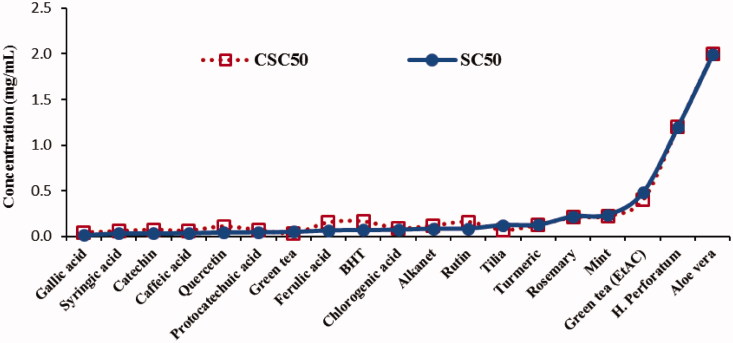
The SC_50_ and CSC_50_ values of standards and extracts (showing a good correlation with R^2 ^=^ ^0.9923).

The 50% radical scavenging values for the plant extracts and standards were observed to correlate well between spectrophotometric method (SC_50_) and the new dropping method (CSC_50_) (Figure 6).

## Conclusions

A suitable plate (TLC plate or chromatography paper), a scanner and an image processing software are sufficient to determine the antioxidant activities with this new method. The new DPPH^•^ method will eliminate the dependence on spectrophotometric instruments. The method is cheaper, simpler, easier and more convenient and can be easily implemented in any environment and situation. The new method has been validated with plant extracts and antioxidant standards with different structures and polarities. Further work needs to be done for the optimisation of the method for various dropping platforms and with highly coloured antioxidant samples.
